# Ultrasound risk marker variability in symptomatic carotid plaque: impact on risk reclassification and association with temporal variation pattern

**DOI:** 10.1007/s10554-020-01801-z

**Published:** 2020-03-06

**Authors:** Isak Stenudd, Elias Sjödin, Emma Nyman, Per Wester, Elias Johansson, Christer Grönlund

**Affiliations:** 1 Department of Public Health and Clinical Medicine, Umeå University, 901 87 Umeå, Sweden; 2 Umeå University, Umeå, Sweden; 3 Department of Pharmacology and Clinical Neuroscience, Umeå University, Umeå, Sweden; 4 Wallenberg Center for Molecular Medicine, Umeå University, Umeå, Sweden; 5 Department of Radiation Sciences, Biomedical Engineering R&D, Umeå University, Umeå, Sweden

**Keywords:** Atherosclerosis, Risk marker, Plaque, Variability, Reclassification

## Abstract

**Purpose:**

Ultrasound examinations of atherosclerotic carotid plaques can be used to calculate risk markers associated with plaque vulnerability. Recent studies demonstrate significant inter-frame variability in risk markers. Here, we investigate risk marker variability in symptomatic plaques and its impact on reclassification of plaque vulnerability, as well as its association with the shape of the temporal variation over the cardiac cycle.

**Methods:**

56 patients with symptomatic carotid stenosis were included in this study. 88 plaques were identified and the plaque risk markers size (area), echogenicity (gray scale median, GSM) and heterogeneity (coarseness) were measured in all frames of ultrasound B-mode image sequences. Inter-frame variability was quantified using the coefficient of variation (CV).

**Results:**

Inter-frame variabilities of the risk markers were area CV 5–8%; GSM CV 4–7%; coarseness CV 8–15% and was in general significantly lower in large as compared to smaller plaques. The variability in GSM risk marker caused a reclassification of vulnerability in 30 to 38% of the plaques. Temporal variations in GSM with a heart rate periodic or drift/trending pattern were found in smaller plaques (< 26 mm^2^), whereas random pattern was found in larger plaques. In addition, hypoechoic plaques (GSM < 25) were associated with cyclic variation pattern, independent of their size.

**Conclusions:**

Risk marker variability causes substantial reclassification of plaque vulnerability in symptomatic patients. Inter-frame variation and its temporal pattern should be considered in the design of future studies related to risk markers.

## Introduction

As one of the leading causes of death and disability worldwide, stroke constitutes a major threat to public health [1]. About 80% of strokes are ischemic and almost one third of these are caused by carotid stenosis, making it one of the most important etiologies [1, 2].

Ultrasonographic techniques are commonly used methods for detection and grading of carotid stenosis in clinical investigation [3, 4]. In order to assess the risk of a cerebrovascular event, ultrasonographic risk markers have been identified. They are typically calculated from a segmented plaque in a single frame of a B-mode ultrasound image sequence. Two such risk markers are the gray scale median (GSM) quantifying the echogenicity of the plaque and the coarseness quantifying the composition heterogeneity of the texture.

Although proven clinically relevant, the risk markers have known issues with sensitivity and specificity for risk prediction [5], and in combination with poor reproducibility, thus emphasizes the importance of methodological improvements [6–8]. Several potential sources of variations have been identified that may influence plaque measurements including the pressure changes during the cardiac cycle where dilation and reduction will cause natural oscillations of the artery diameter [9], respiration and movement of the probe.

Previous research has shown significant inter-frame variability in GSM (and other risk markers) in ultrasound image sequences during the cardiac cycle [10–12] in both asymptomatic and symptomatic plaques. The variability was shown to be associated with plaque size and echogenicity but with somewhat contradictory results [10, 11]. The impact of this variability was also shown to cause a 20% risk re-classification in asymptomatic plaques (small plaques, early disease) [11].

However, this risk re-classification remain to be explored for symptomatic plaques (large plaques, late disease). Also, while significant differences in GSM have been shown between systolic and diastolic phases [11, 12], the shape of the temporal pattern (e.g. cyclic, random) of the risk marker variation has not been explored. Taken together, these issues may contribute to the understanding of the impact of the variability throughout the different stages of the disease, and how risk marker assessment should be improved regarding reproducibility and sensitivity.

The aim of this study was to investigate risk marker variability in participants with symptomatic carotid plaques and assess the impact of variability on risk re-classification, and the association between variability and temporal characteristics of the risk markers (e.g. cyclic or random). In addition, we evaluated the influence of size and echogenicity on the risk marker variability.

## Materials and methods

### Study design and subjects

In this study, patients were retrospectively selected from the prospective cohort study of ANSYSCAP (Additional Neurological Symptoms before Surgery of the Carotid Arteries—a Prospective study) [4]. We selected subjects from the study material of 230 patients with symptomatic carotid stenosis based on the criteria of digitally stored cine loops (n = 143) and sufficient image quality. 59 of the digitally stored examinations were performed using the Color Doppler technique in all the sequences, making analysis of the beat-to-beat variations impossible since the Doppler-signal covered parts of the plaques during systole. From the remaining 84 examinations, another 28 were excluded due to absence of visible plaques, poor image quality, plaque shadows or excessive movement of the probe during the sequence. The remaining 56 patients were included in our study and a total of 88 plaques were analyzed.

The ANSYSCAP-study, making its inclusion of patients between 1 August 2007 and 31 December 2009, was reviewed by the regional ethics committee at Umeå University before it was conducted. They concluded that the study did not require committee approval since it was strictly observational.

### Ultrasound acquisition

The examinations were performed by experienced ultrasonographers, following the protocol of standard US clinical settings, using a conventional system (Acuson Sequoia 512®, Siemens Company, Mountain View, CA) with a 8L5 linear transducer, projecting towards the plaque in a longitudinal view. The mean duration of the B-mode sequences was 2.0 s and the frame rate ranged from 12 to 26 Hz.

### Risk marker calculation

Based on the framework originally described by Nicolaides et al. [13], a plaque texture analysis software (PLAQ, Department of Biomedical Engineering R&D, Västerbotten County Council, Umeå, Sweden), that has been described in earlier publications by our research group [11, 14, 15], was used to calculate risk markers by the sequential steps of image normalization and manual delineation of plaques.

In short, a plaque was manually delineated in an B-mode image, and then two region of interests (ROIs) were manually selected in the darkest area of the lumen and the brightest area of the adventitia, respectively. The image was then automatically normalized to 190 in the adventitia and 0 in the lumen based on the intensities of the two ROIs, and pixel density was standardized to 20 pixels/mm [11, 13]. The procedure was repeated in every frame of the ultrasound sequence in order to estimate risk marker variation. All plaque analysis was performed by a single operator (E.S).

The risk markers chosen for variability analysis were measurements of echogenicity (GSM), size (area), plaque type and heterogeneity (coarseness). The risk markers were analyzed in longitudinal images, according to guidelines [16], and the variables were chosen because they have shown a strong correlation with plaque symptomatology [5, 13]. GSM is the median grey-scale value of all the pixels in the plaque [13], where low values (< 25 to 32) are associated to symptomatic plaques and high values to asymptomatic plaques (e.g. [5, 20].). The coarseness is a measurement of granularity, i.e. how fine-grain or coarse-grain a structure is, and was calculated using the neighborhood gray-tone difference matrix. Low values (< 15) are associated with heterogenic composition and vulnerable plaques [5, 17]. Lastly, the plaque type divides the plaques into four groups based on their echodensity, according to the modified Geroulakos classification [18] depending on the percentage of pixels in the plaque area with GSM values > 25. Type 1 = uniformly echolucent (< 15% with GSM values > 25); type 2 = mainly echolucent (15–50%); type 3 = mainly echogenic (50–85%); and type 4 = uniformly echogenic (> 85%) [10, 13].

In addition, an un-normalized GSM was calculated [10, 11] as the median echo intensity of all the pixels in each segmented plaque image, without the echo intensity normalization described above. This was done in order to analyze the variability of echogenicity in plaques without being influenced by the normalization procedure and echogenicity changes in blood or adventitia.

### Statistical analysis

Statistical analysis was carried out using MATLAB R2015a (The MathWorks, Inc., Natick, Massachusetts, USA).

**Fig. 1:**
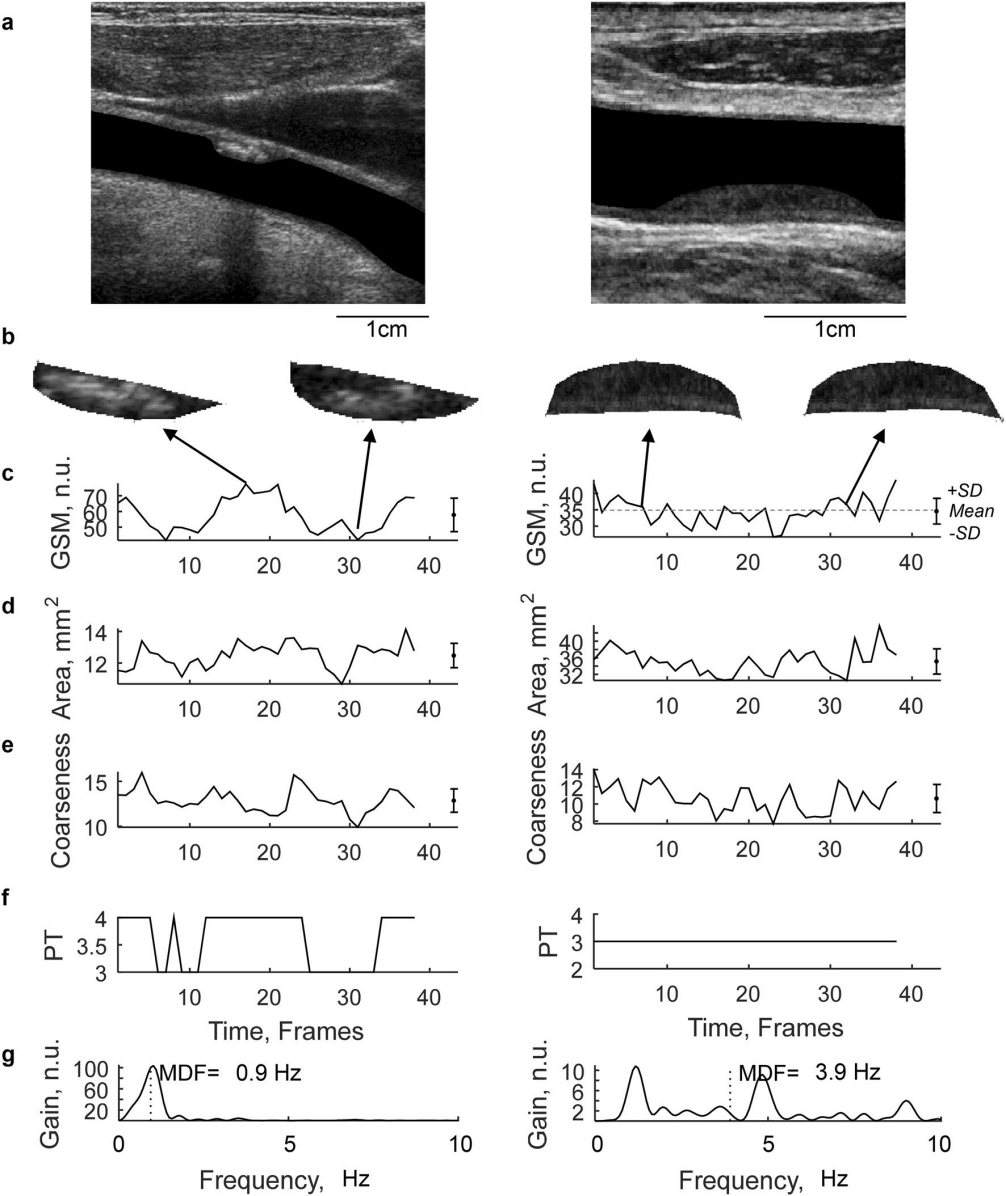
Examples of results from two plaques. **a** Longitudinal B-mode projection of the carotid with plaque, **b** cropped B-mode plaque images at two different time points during the image sequence, Temporal variations throughout the image sequence for **c** GSM, **d** area, **e** Coarseness and **f** Plaque Type (PT). **g** The power spectrum of the GSM signal (**c**) and the estimated Median Frequency (MDF). The sequence corresponds to about 2 s (Frame rate 20 Hz). Mean values and standard deviation (SD) were computed for each sequence for GSM, Area and Coarseness (**c**–**e**) as indicated in **c**

Risk marker variability was quantified by computing the coefficient of variation (CV) for every risk marker in all of the frames constituting a plaque image sequence. Further, the temporal pattern of the variability was assessed using the median frequency descriptor (MDF), calculated as the frequency that divides the power spectrum of the GSM variability signal in equal halves (see Fig. 1g) and has the units 1/s or Hz. A high MDF value correspond to a pattern that has fast changes in frame-to-frame variation (random) and a low value correspond to slow changes such as heart rate periodic pattern or pattern with drift/trend.

Furthermore, to determine how the variability is influenced by plaque size, echogenicity and temporal pattern, the collection of values for area, GSM and MDF, respectively, were each divided at the median into dichotomized groups. Then, an unpaired, nonparametric Mann–Whitney *U*-test was used to assess whether the CV of the risk markers differed between the dichotomized groups.

Lastly, the effect of variability on vulnerability reclassification was investigated by counting the number of plaques that changed from plaque type ≤ 2 to ≥ 3 or crossed specific threshold levels in GSM. Consequently, for analysis of plaque type the cut-off value was ≤ 2 [19], and the two cut-off values of < 32 and < 24 for GSM were chosen for analysis of range-dependent reclassification [5, 20]. The reclassification accuracy was estimated by the standard deviation of random sampling with replacement. The *p* value was set to 0.003 by Bonferroni correction (0.05/16) because of multiple comparisons.

### Inter- and intra-rater reliability

In order to assess inter-rater reliability, two operators (E.S. and E.N.) independently carried out analysis of 20 randomly selected plaque image sequences. Intra-rater reliability, on the other hand, was evaluated by the intra-class correlation coefficient (ICC). With a confidence interval of 95%, ICC was measured by letting one operator analyse 20 randomly selected sequences twice. The time that had passed before reanalysis was six months.

## Results

Characteristics of the 56 included subjects are summarized in Table 1. Figure 1 shows examples of results from two plaques and their calculated risk markers of their image sequences. The results of the risk marker measurements of the 88 analyzed carotid plaques is summarized in Table 2.

**Table 1:** Baseline characteristics of the subjects

Patient characteristics	N = 56 (%)
Age, mean (SD; range), years	69.7 (8.5; 47–85)
Women	22 (39.3)
Systolic blood pressure ≥ 140 and/or diastolic ≥ 90 mmHg	37 (66.1)
Current smoker	11 (19.6)
Diabetes	13 (23.2)
Previous stroke	8 (14.3)
Previous myocardial infarction	13 (23.2)
Congestive heart failure	2 (3.6)
Any type of anti-platelet or anti-coagulation medication	56 (100)
Any type of blood pressure reducing medication	52 (92.9)
Any type of lipid-lowering medication	51 (91.1)
50–69% stenosis	10 (17.9)
70–99% stenosis	43 (76.8)
Near occlusion	3 (5.4)
Stroke as presenting event	31 (55.4)
TIA as presenting event	13 (23.2)
Amaurosis fugax as presenting event	8 (14.3)
Retinal artery occlusion as presenting event	4 (7.1)

**Table 2:** Measurements of plaques: overall results and comparison based on size, echogenicity and variation characteristic

		Size (area, mm^2^)			Echogenicity (GSM, n.u.)			Variability pattern (MDF, Hz)		
	All	< 30.2	> 30.2	p	< 39	> 39	p	< 1.3	> 1.3	p
*N*	*88*	*44*	*44*		*44*	*44*		*44*	*44*	
Area	32.9 (17.7)	17.9 (5.30)	48.0 (12.0)		34.6 (18.2)	31.3 (17.3)	0.380	25.7 (14.5)	40.2 (17.9)	0.000
CV	0.07 (0.03)	0.08 (0.03)	0.05 (0.02)	0.000	0.07 (0.03)	0.07 (0.03)	0.791	0.07 (0.03)	0.06 (0.03)	0.041
GSM	44.1 (22.2)	46.5(23.4)	41.7(21.0)	0.348	28.1 (7.9)	60.1 (20.4)		45.9 (21.1)	42.30 (23.4)	0.457
CV	0.12 (0.06)	0.13 (0.07)	0.10 (0.05)	0.021	0.14 (0.07)	0.10 (0.05)	0.003	0.13 (0.07)	0.10 (0.05)	0.040
GSM RAW	53.6 (17.2)	55.8 (16.0)	51.4 (18.2)	0.211	44.6 (14.1)	62.6 (15.2)		54.7 (17.9)	52.5 (15.5)	0.538
CV	0.06 (0.04)	0.07(0.05)	0.04(0.02)	0.001	0.06 (0.04)	0.05 (0.04)	0.668	0.07 (0.05)	0.04 (0.02)	0.000
Coarseness	13.7 (4.6)	13.6(5.0)	13.8(4.2)	0.886	11.4 (4.1)	16.0 (3.9)	0.000	13.6 (4.3)	13.8 (5.0)	0.881
CV	0.12 (0.09)	0.12 (0.10)	0.11 (0.08)	0.520	0.15 (0.11)	0.08 (0.04)	0.000	0.12 (0.09)	0.11 (0.08)	0.561
MDF GSM	1.6 (1.1)	1.3 (0.8)	1.9 (1.2)	0.006	1.8 (1.2)	1.4 (0.9)	0.226	0.8 (0.2)	2.4 (1.0)	

Plaque area ranged from 8.9 to 78.0 mm^2^, GSM from 5.6 to 130.9, un-normalized GSM from 8.0 to 93.7, coarseness from 2.8 to 26.5 and MDF from 0.4 to 5.9 Hz. The variabilities for area, GSM, un-normalized GSM and coarseness were, on average, 6.6%, 11.8%, 5.6% and 11.6% respectively.

### Influence of size

The CV for measurements of area were significantly higher for small plaques (< 30 mm^2^, 8%) than for large plaques (> 30 mm^2^, 5%) (*p* < 0.001). No significant differences in the CV for GSM were found between small and large plaques. However, for un-normalized GSM, the CV value was significantly higher for small plaques (7%) than for large plaques (4%) (*p* = 0.001) (Table 2).

### Influence of echogenicity

There was no significant difference in CV of GSM between echogenic and echolucent plaques, even though it was higher for echolucent plaques (GSM < 39, 14%) than for echogenic plaques (GSM > 39, 10%) (*p* = 0.003). Regarding coarseness, however, the mean value was higher for echogenic plaques (16) than for echolucent plaques (11) (*p* < 0.001). Moreover, the CV for coarseness was higher for echolucent plaques (15%) than for echogenic plaques (8%) (*p* < 0.001) (Table 2).

### Association with temporal variation pattern

Plaques with higher median frequency (MDF > 1.3 Hz, corresponding to fast variations of the temporal pattern) had significantly larger area as compared with plaques with MDF < 1.3 Hz, corresponding to slow changes in the temporal patterns (area 40 vs 26 mm^2^, p < 0.001).

In addition, plaques with higher MDF had significantly lower CV for un-normalized GSM as compared to plaques with lower MDF (CV 4% vs 7%, p < 0.001, for MDF > 1.3 vs MDF < 1.3) (Table 2).

**Fig. 2:**
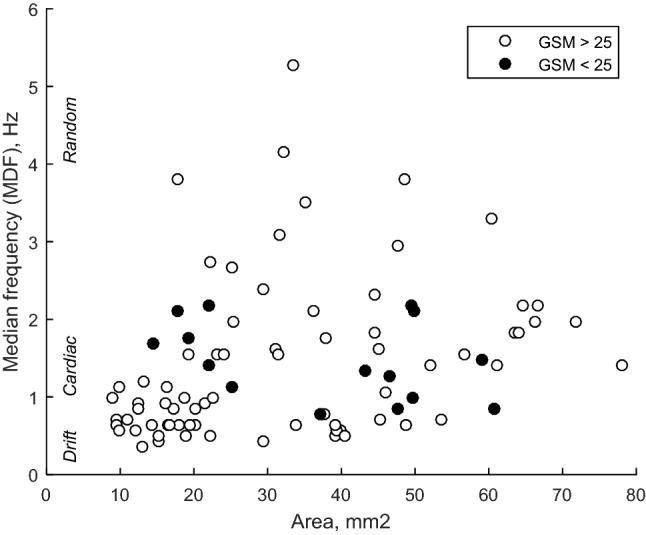
Median frequency (MDF) of the GSM variation vs plaque area. A low MDF implies heart rate cyclic or temporal drift/trend, whereas a high MDF implies random variation of the GSM. Low echogeneic plaques (GSM < 25, potentially vulnerable) had around 1–2 Hz MDF for both large and small plaques

Moreover, hypoechoic plaques with GSM < 25 had a MDF of their temporal variation pattern in the range 1–2 Hz independent of size, as compared to plaques with GSM > 25 (Fig. 2). The result indicate that hypoechoic plaques (GSM < 25) have more cyclic temporal variation patterns related to cardiac pacing, independent of their size.

### Effect on reclassification

**Fig. 3:**
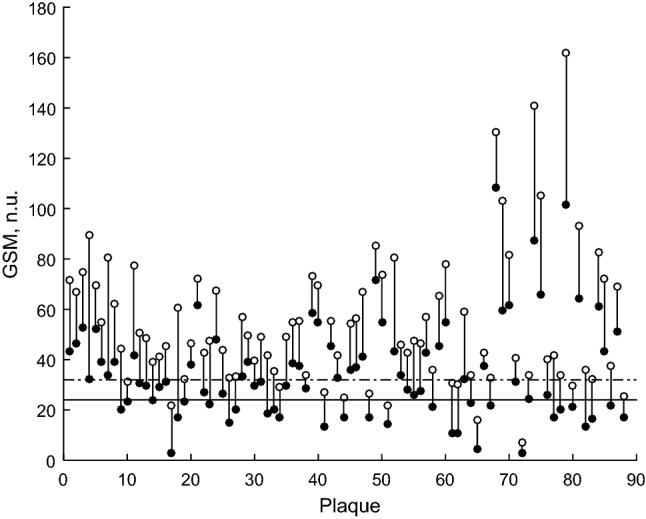
Illustration of reclassification based on maximum (open circle) and minimum (filled circle) values for measured gray-scale median on 57 carotid plaques. Reclassification is defined by a crossing cutoff value of 24 (*solid line*) (Christodoulou et al. [5]) or 32 (*dotted line*) (El-Barghouty et al. [20]). *GSM *gray-scale median

Figure 3 illustrates the GSM variation range throughout the ultrasound sequence, i.e. the cine loop, for each plaque. 38% (n = 33) of the analyzed plaques were reclassified at cutoff in GSM = 32 and 30% (n = 26) were reclassified at GSM = 24. In addition, 31% of plaques changed plaque type between type 3 and 2 in one or more of the frames constituting the cine loop.

The mean probability of reclassification concerning all of the plaques that in one or more frame crossed the cutoff value of 24 or 32 was 18% or 14%, respectively. Moreover, the probabilities ranged from 2.6% to 47% when analyzing the plaques crossing the cutoff 24, and between 2.6% and 39% for the cutoff 32, respectively.

### Inter- and intra-rater reliability

The intra-rater reliability ICCs for area, GSM and coarseness were 0.93 (95% CI 0.87–0.95), 0.93 (95% CI 0.88–0.96) and 0.83 (95% CI 0.71–0.91), respectively. The inter-rater reliability ICCs for area, GSM and coarseness were 0.71 (95% CI 0.50–0.81), 0.72 (95% CI 0.49–0.87) and 0.83 (95% CI 0.68–0.92), respectively.

## Discussion

We investigated the frame-by-frame variations of risk markers for carotid plaques in symptomatic patients and its influence on plaque reclassification and shape of temporal variations (fast, slow changes). The variability of plaque risk markers within the ultrasound sequences was found to range from 6 to 12% and caused reclassification of plaque vulnerability in 30% or 38% of cases, depending on cutoff value.

### Impact on vulnerability classification

The impact of the variability in GSM on risk classification showed that 30% or 38% of plaques were reclassified, having at least one frame with GSM value crossing the classification cut-off value. This is similar to a previous study by our group on asymptomatic plaque variability causing 16–25% reclassification [11]. To quantify how often GSM crossed the cut-off value, the probability of reclassification (i.e. crossing the cutoff value) was calculated. This probability was on average 14% to 18%, depending on the cutoff value, which supports that the re-classification is not caused by single “outlier” values of the GSM during the sequence. The probability of reclassification introduced here could be a more robust tool to analyze the impact on vulnerability re-classification.

### Association with temporal variation pattern

Rapid changes in temporal variation (high MDF) with lower amplitude (CV of un-normalized GSM) was found in large plaques as compared with small plaques that had slower temporal variations and higher amplitude (Table 1). This indicates that smaller plaques have a cyclic variability caused by heart rate periodic pattern or pattern with drift/trend, whereas the variability of risk markers of large plaques are more random. This supports the hypothesis stated in previous research that variation may be caused by out-of-plane motion or compression during the cardiac cycle; implying that small plaques may easier move out-of-plane than large ones.

Hypoechoic plaques with GSM < 25 had a low MDF independent of their size. The cutoff value originates from the modified Geroulakos classification [18], which is used to assess plaque vulnerability. This study included plaques from symptomatic individuals. Hypoechoic plaques are generally considered to be more vulnerable and more prone to deformation caused by variation in blood pressure [21, 22]. Consequently, our results indicate that vulnerable plaques show a slow cyclic temporal variation in measurement of echogenicity, which is independent of their size.

### Influence of size

Our results show that smaller plaques had a higher variability in plaque area than larger plaques. This is similar to the results of a previous study by Nyman et al. [11] but contrasting to a study by Kanber et al. [10]. The former study suggested that the higher variability, not seen by Kanber et al. could be due to the fact that the plaques included in the Nyman et al. study had a smaller mean plaque area. However, in this study, we found variability despite we have the same mean plaque area as Kanber et al. The increased variability may either be due to a greater sensitivity of small plaques to manual delineation or out-of-plane motion. As the variability for un-normalized GSM also increased for small plaques, the theory that variation is caused by out-of-plane motion is supported in front of unprecise manual delineation. This is because the latter would not essentially influence the measurement of echogenicity.

Moreover, the difference in variability for un-normalized GSM could not be seen for normalized GSM. This could be caused by the propagation of errors during the many steps included in the risk marker assessment [23].

The results of this study on symptomatic plaques together with a previous study by our group on asymptomatic plaques [11], as well as other studies (e.g. Ostling et al. 2007) [24], show that smaller plaques have a higher variability in risk marker measurements, and supports the thesis that including plaques larger than a certain cut-off would result in more stable estimates of the risk markers.

### Influence of echogenicity

Although there was not a strict significant difference (*p* = 0.003 compared to limit *p* = 0.0025), echolucent plaques had a higher variability in GSM than echogenic plaques. This could be explained by the fact that echogenic plaques are less elastic due to their composition of fibrotic and calcified tissues, and echolucent plaques are more elastic due to the lipid-rich composition and may deform easier by variations in blood pressure during the cardiac cycle [21, 22]. Difficulties in manual delineation of hypoechoic plaques could also have caused the higher variability. However, the latter would imply a corresponding higher variability in plaque area for echolucent plaques. No such difference could be found. In addition, the variability of coarseness was significantly higher for echolucent plaques. Heterogeneity variations could, as well as changes in acoustic impedance influencing GSM, be due to deformation or strain of the plaque during the cardiac cycle.

Furthermore, we found that the mean value for coarseness was significantly lower for echolucent plaques than for echogenic plaques, indicating that echolucent plaques had a more heterogenous composition.

### Limitations

Our selection of examinations from the ANSYSCAP-study was limited by the difficulties of analyzing ultrasound sequences due to acoustic shadows or Doppler signals covering the plaques, poor image quality or technical obsolescence in respect to data storage. In addition, the acquired data was not optimized for studying variability since the sequences were short and lacked a corresponding ECG.

Furthermore, a limitation with the 2-D-ultrasound technique is that it is sensitive to out-of-plane motion. Using 3-D-ultrasound technique could potentially be an improvement since the whole plaque volume could be assessed in order to calculate risk markers [25].

The manual segmentation of the plaque may have influenced our results on the intra-rater level. However, the calculated value for intra-rater reliability indicates that the manual steps had a low impact on our results. Inter-rater reliability was lower than intra-rater reliability, but similar to that reported by others [13].

## Conclusions

Risk marker variability is substantial in ultrasound image sequences of plaques in symptomatic patients, and may cause substantial reclassification of plaque vulnerability. The temporal variation pattern of the risk markers (fast changes compared with slow changes) is indicated to be related to plaque vulnerability. Inter-frame variation and its temporal pattern should be considered in the design of future studies related to risk markers.

## Data Availability

The data that support the findings of this study are available from the ANSYSCAP-study but restrictions apply to the availability of these data, which were used under license for the current study, and so are not publicly available. Data are however available from the authors upon reasonable request and with permission of the co-authors Per Wester and Elias Johansson (owners of the ANSYSCAP data).
